# Supplementary material for: The interactome of intact mitochondria by cross-linking mass spectrometry provides evidence for coexisting respiratory supercomplexes
Source: Mol Cell Proteomics. 2017 Dec 8;17(2):216–32. doi: 10.1074/mcp.RA117.000470 (PMC5795388; doi:10.1074/mcp.RA117.000470)
Supplement: Supplemental Data [file supp_RA117.000470_133922_0_supp_23978_fzffwf.zip › spectra_annotation/mito_spectra_annotation/139-1-1-1-17-1.pdf]

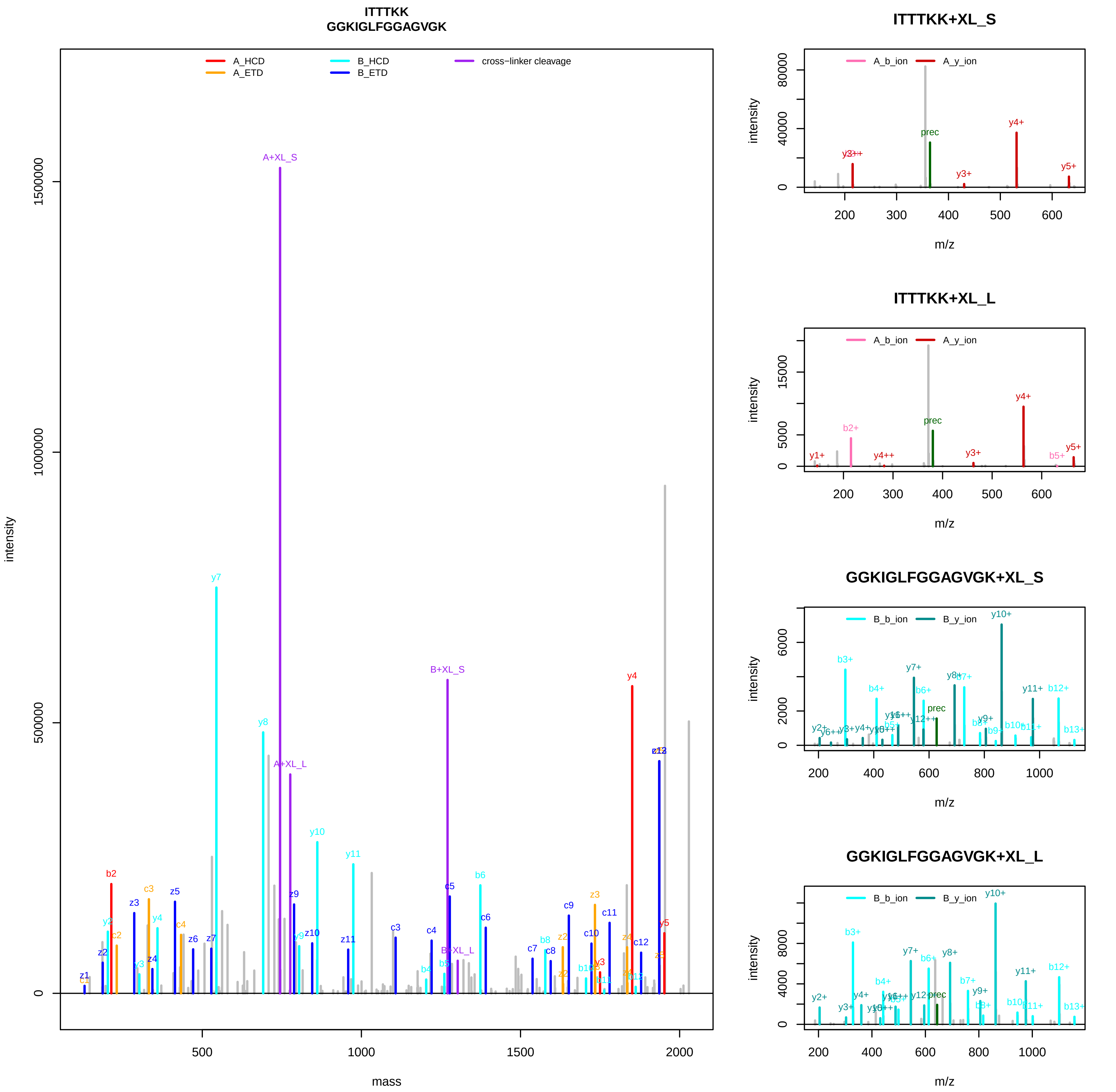
ITTTKK
GGKIGLFGGAGVGK
A_HCD
A_ETD
B_HCD
B_ETD
cross−linker cleavage
intensity
mass
0
500000
1000000
1500000
500
1000
1500
2000
A+XL_S
B+XL_S
A+XL_L
B+XL_L
y7
y8
y10
y11
y4
y5
y3
b2
c2
c3
c4
z2
z3
z4
z5
z6
z7
z9
z10
z11
z12
c5
c6
c7
c8
c9
c10
c11
c12
b6
b8
ITTTKK+XL_S
ITTTKK+XL_L
GGKIGLFGGAGVGK+XL_S
GGKIGLFGGAGVGK+XL_L
A_b_ion
A_y_ion
B_b_ion
B_y_ion
prec
m/z
intensity
y4+
y5+
y3+
b2+
b5+
y1+
y10+
y11+
y7+
y8+
b3+
b4+
b6+
b7+
b12+
b13+
